# *Candida* Enteritis Diagnoses in the United States: Prevalence and Clinical Insights from a Large Commercial Insurance Database

**DOI:** 10.1007/s10620-026-09905-7

**Published:** 2026-04-16

**Authors:** Kaitlin Benedict, Jeremy A. W. Gold, Mark Pimentel

**Affiliations:** 1 Mycotic Diseases Branch, Division of Foodborne, Waterborne, and Environmental Diseases, National Center for Emerging and Zoonotic Infectious Diseases, Centers for Disease Control and Prevention, 1600 Clifton Road NE, Mailstop H24-9, Atlanta, GA USA; 2 Medically Associated Science and Technology Program, Cedars-Sinai Medical Center, Los Angeles, CA USA

**Keywords:** Candida, Fungi, Yeasts, Enteritis, Intestine, small, Insurance, Health

## Abstract

**Background:**

*Candida* overgrowth in the small intestine can cause non-specific gastrointestinal symptoms mimicking those of other conditions, making diagnosis challenging.

**Aims:**

To describe the characteristics of patients with *Candida*-related intestinal illness in a large US commercial health insurance claims dataset.

**Methods:**

We used the Merative™ MarketScan® Commercial/Medicare Database to identify patients who received *Candida* enteritis diagnosis codes.

**Results:**

We identified 3,710 patients. Rates were higher among women (20.3/100,000), patients ages 35–44 (19.3/100,000) and in the West (28.1/100,000). Fatigue (44%) and abdominal pain (26%) were common symptoms. Few patients (6%) received an esophagogastroduodenoscopy, the preferred diagnostic method, and 38% received systemic antifungal therapy.

**Conclusions:**

Increased healthcare provider awareness about proper diagnosis and treatment of intestinal *Candida* overgrowth could be beneficial.

**Supplementary Information:**

The online version contains supplementary material available at https://doi.org/10.1007/s10620-026-09905-7.

## Introduction

*Candida* species are normal inhabitants of human mucous membranes but can overgrow under certain conditions such as immunosuppression, antibiotic use, and other microbiome disruption. Small intestinal fungal overgrowth (SIFO) refers to overgrowth of fungi (typically *Candida*) in the small intestine, which can cause symptoms including abdominal pain, bloating, and diarrhea [1]. These symptoms overlap with those of other gastrointestinal conditions, including small intestinal bacterial overgrowth (SIBO), which is more widely recognized and understood than SIFO [2]. Given the non-specific symptoms of these conditions, diagnosis can be challenging. Consequently, clinical and epidemiological data about *Candida* overgrowth in the gastrointestinal tract are scarce. We analyzed a large US commercial health insurance claims dataset to describe the demographic and clinical characteristics of patients with *Candida*-related intestinal illness.

## Methods

The Merative™ MarketScan® Commercial Database contains longitudinal employer-sponsored private health insurance claims data including Medicare Supplemental and Medicare Advantage plans from > 65 million persons throughout the United States during 2017–2024 (https://www.merative.com/documents/brief/marketscan-explainer-general). We identified patients with any International Classification of Diseases, Tenth Revision, Clinical Modification (ICD-10-CM) diagnosis code for *Candida* enteritis (B37.82) not listed on a laboratory or imaging claim alone during 7/1/2017–9/30/2024 (study window). ICD-10-CM code B37.82 was the only available code related to candidal intestinal illness at the beginning of the study window. The index date was the date the diagnosis code was first used during the study window. We selected patients with continuous insurance enrollment during the − 180 to + 30 days around the index date and examined demographic characteristics, selected underlying conditions, signs and symptoms, diagnostic tests, healthcare provider type, and antifungal treatment (Supplemental Table 1). We calculated prevalence of *Candida* enteritis diagnoses and used logistic regression to evaluate associations between each selected underlying condition and having a *Candida* enteritis diagnosis code. The comparison group was patients who did not have a *Candida* enteritis diagnosis code and had continuous insurance enrollment during the − 180 to + 30 days around their first healthcare visit in the study window.

## Results

In total, 3710 patients with *Candida* enteritis diagnosis codes met the enrollment criteria (prevalence: 14.3 per 100,000 patients) (Table 1). Patients’ median age was 44 years (interquartile range [IQR] 31–56); prevalence was highest among patients ages 35–44 years (19.3/100,000), female patients (20.3/100,000), and in the West (28.1/100,000).

**Table 1:** Characteristics of patients with ICD-10-CM diagnosis code B37.82 for *Candida* enteritis — United States, 7/1/2017 to 9/30/2024

	n	%	Denominator	Rate per 100,000 patients
Total	3710		25,873,638	14.3
Demographic characteristics				
Median age in years (IQR)	44.0	(31.0–56.0)		
Age group in years				
< 18	424	11.4	5,413,185	7.8
18–34	703	18.9	6,385,283	11.0
35–44	744	20.1	3,851,806	19.3
45–54	793	21.4	4,143,496	19.1
55–64	804	21.7	4,183,876	19.2
≥ 65	242	6.5	1,895,992	12.8
Sex				
Male	1023	27.6	12,646,496	8.1
Female	2687	72.4	13,227,142	20.3
Urban/rural classification (n = 2,844)				
Non-rural	2471	86.9	18,271,970	13.5
Rural	373	13.1	2,913,845	12.8
Region^1^ (n = 3,609)				
Northeast	472	13.1	3,992,360	11.8
Midwest	622	17.2	5,509,842	11.3
South	1315	36.4	10,264,894	12.8
West	1,200	33.3	4,271,645	28.1
Signs and symptoms on or in the 180 days before index date				
Abdominal distention	553	14.9		
Abdominal pain	949	25.6		
Abnormal weight gain	203	5.5		
Abnormal weight loss	106	2.9		
Constipation	553	14.9		
Diarrhea	408	11.0		
Fatigue or malaise	1623	43.7		
Insomnia	386	10.4		
Nausea or vomiting	368	9.9		
Outpatient prescription medications in the 180 days before index date				
Systemic antibacterials	1231	33.2		
Proton pump inhibitors	407	11.0		
Diagnostic testing on or in the 180 days before index date				
Fungal stool culture	284	7.7		
Bacterial stool culture	461	12.4		
*Candida* antibody test	456	12.3		
Breath hydrogen or methane test	70	1.9		
Esophagogastroduodenoscopy	223	6.0		
Healthcare setting on the index date^2^				
Outpatient	3669	98.9		
Inpatient	54	1.5		
Emergency department	15	0.4		
Provider type on the index date^2^				
Family practitioner or internal medicine	1632	44.0		
Alternative therapist	430	11.6		
Nurse practitioner or physician assistant	344	9.3		
Laboratory	271	7.3		
Acute care hospital	175	4.7		
Chiropractor	147	4.0		
Gastroenterologist	74	2.0		
Infectious disease specialist	9	0.2		
None of the above provider types	937	25.3		
Antifungal treatment in the 7 days before to 30 days after index date^3^	1398	37.7		
Fluconazole	871	23.5		
Nystatin tablets	725	19.5		
Other systemic antifungal	34	0.9		

IQR = interquartile range

^1^Based on residence of primary insurance beneficiary

^2^Non-mutually exclusive category

^3^Median fluconazole days’ supply: 18 (IQR 10–30); Median nystatin days supply: 30 (IQR 21–30). Other systemic antifungals included: isavuconazole (n = 2), itraconazole (n = 25), posaconazole (n = 3), and voriconazole (n = 4)

Fatigue (44%), abdominal pain (26%), constipation (15%), and abdominal distention (15%) were among the most frequently recorded symptoms. Common selected underlying conditions included anxiety disorder (25%), vitamin D deficiency (24%), and hypothyroidism (22%), and 33% previously received systemic antibacterial medication. Adjusting for age, sex, and region, patients with *Candida* enteritis diagnosis codes were significantly more likely than the comparison group (n = 25,871,280) to have diagnosis codes for bacterial intestinal tract infection (aOR [adjusted odds ratio]: 495.50, 95% confidence interval [CI]: 427.48–574.35), Lyme disease (aOR: 64.39, CI: 54.80–75.66), iron deficiency (aOR: 29.17, CI: 25.11–33.89), irritable bowel syndrome (aOR: 27.66, CI:25.19–30.37), and *Helicobacter pylori* (aOR: 25.02, CI: 19.88–31.49) (Table 2). Patients with *Candida* enteritis diagnosis codes had a higher median number of healthcare visits in the pre-index period vs. the comparison group (9 [IQR: 4–17] vs. 1 [IQR: 0–4], p < 0.001).

**Table 2:** Other diagnoses associated with ICD-10-CM diagnosis code B37.82 for *Candida* enteritis — United States, 7/1/2017 to 9/30/2024

Underlying conditions on or in the 180 days before index date	n	%	aOR^1^	(95% CI)
Anxiety disorder	933	25.1	4.96	(4.60–5.35)
Autoimmune inflammatory disease	294	7.9	4.41	(3.90–4.99)
Bacterial intestinal tract infection	215	5.8	495.50	(427.48–574.35)
Chronic pain	223	6.0	3.13	(2.73–3.60)
Colectomy	48	1.3	4.33	(3.24–5.80)
Depression	519	14.0	3.26	(2.97–3.59)
Diabetes	300	8.1	1.22	(1.08–1.39)
Dietary counseling and surveillance	198	5.3	3.15	(2.72–3.65)
Dyslipidemia	874	23.6	2.09	(1.91–2.28)
Fibromyalgia	143	3.9	7.16	(6.04–8.49)
Gastroesophageal reflux disease	550	14.8	3.75	(3.42–4.14)
*Helicobacter pylori*	76	2.0	25.02	(19.88–31.49)
Hypertension	617	16.6	1.07	(0.97–1.18)
Hypothyroidism	815	22.0	5.03	(4.62–5.48)
Immunosuppression	256	6.9	2.09	(1.83–2.39)
HIV	12	0.3	2.82	(1.60–4.98)
Cancer	134	3.6	1.57	(1.31–1.89)
Immunosuppressive medication besides prednisone^2^	61	1.6	3.00	(2.33–3.87)
Prednisone (≥ 21 days’ supply)	72	1.9	3.23	(2.54–4.12)
Solid organ or stem cell transplant	21	0.6	3.31	(2.15–5.09)
Iron deficiency	185	5.0	29.17	(25.11–33.89)
Irritable bowel syndrome	561	15.1	27.66	(25.19–30.37)
Lyme disease	169	4.6	64.39	(54.80–75.66)
Malnutrition	58	1.6	10.34	(7.88–13.56)
Nutritional anemia	308	8.3	5.36	(4.75–6.05)
Overweight and obesity	387	10.4	1.96	(1.76–2.18)
Smoking (current or past)	129	3.5	1.17	(0.97–1.40)
Urinary tract infection	209	5.6	1.91	(1.65–2.21)
Vitamin D deficiency	892	24.0	6.62	(6.11–7.17)
Vulvovaginal candidiasis	114	3.1	6.32	(5.22–7.65)

^1^aOR: adjusted odds ratio, adjusted for age, sex, and region

^2^TNF-alpha inhibitors, IL-17 and IL-23 inhibitors, mycophenolate mofetil, and tacrolimus

Most patients with *Candida* enteritis diagnosis codes visited outpatient settings on the index date (99%), specifically family practice or internal medicine providers (44%) or alternative therapists (12%); 2% visited a gastroenterologist. Esophagogastroduodenoscopy was performed in 6% of patients. Total, 38% received antifungal treatment, including fluconazole (24%) and nystatin tablets (20%).

## Discussion

This exploratory analysis of commercial health insurance data revealed that *Candida* enteritis diagnosis codes were assigned primarily to middle-aged, immunocompetent female outpatients, which contrasts with limited literature characterizing *Candida* enteritis as a condition affecting older, immunosuppressed, hospitalized patients [3]. These findings suggest that some patients who received a *Candida* enteritis diagnosis code might actually have had a different condition, such as suspected SIFO.

Patients with *Candida* enteritis diagnosis codes more frequently had anxiety, irritable bowel syndrome, and Lyme disease diagnoses, and had higher rates of healthcare utilization vs. the comparison group, suggesting a population seeking care for unexplained or inadequately controlled gastrointestinal symptoms, notably abdominal pain and distension. The high adjusted odds ratios observed for bacterial intestinal tract infection and Lyme disease may reflect residual confounding or coding practices rather than true clinical associations and should be interpreted with caution. The higher rate of *Candida* enteritis diagnoses in the West could reflect increased awareness of intestinal *Candida* overgrowth among healthcare providers, particularly those practicing alternative medicine. This finding aligns with the region’s higher prevalence of complementary health approaches, particularly among patients with chronic health conditions [4, 5].

Although 38% of patients received fluconazole or nystatin tablets, few saw a gastroenterologist (2%) or received an esophagogastroduodenoscopy (6%), highlighting the uncertainty of candidal enteritis or SIFO diagnoses in this dataset. Formal clinical practice guidelines for diagnosing and treating SIFO are lacking, but the most definitive diagnostic method is jejunal aspirate culture [1]. Our study might overestimate true disease prevalence due to incorrect diagnosis or underestimate prevalence due to lack of clinician awareness and miscoding. A new ICD-10-CM code for SIFO was introduced in October 2023, presenting opportunities for better coding precision. This code was rarely used in the analyzed dataset (< 50 patients in the year following its introduction), limiting our ability to characterize those patients. The low number of SIFO diagnoses could reflect the limited time frame for adoption of the new diagnosis code and lack of clinician awareness and standardized diagnostic criteria for SIFO.

Although MarketScan data are broadly representative of patients with commercial health insurance, they do not necessarily represent patients with other health insurance types or without insurance. Misclassification is inherent in claims-based data (i.e., *Candida enteritis* vs. SIFO), and symptom under-coding is likely. Lastly, MarketScan data do not contain information about over-the-counter medications, dietary supplements, testing not covered by insurance, or laboratory results.

Our results provide preliminary data on *Candida*-related intestinal illnesses and baseline suspected prevalence estimates among commercially insured patients in the United States. The findings underscore the importance of efforts to help increase recognition and understanding of these conditions and the need for standardized guidelines for SIFO diagnosis and management. Future work could investigate the prevalence of confirmed SIFO and examine associations between antifungal treatment and symptom resolution among patients with SIFO.

## Supplementary Information

Below is the link to the electronic supplementary material.Supplementary file1 (DOCX 19 KB)

## Data Availability

This study used third-party data that we cannot legally distribute. All relevant summary data are within the manuscript and the supporting files. The raw data underlying the results presented are available from the Merative TM MarketScan® Research Databases: [https://www.merative.com/documents/brief/marketscan-explainer-general]. Others can access the data by going to this website and contacting Merative TM. The authors did not have any special access privileges that others would not have.
