# Supplementary material for: Candida Enteritis Diagnoses in the United States: Prevalence and Clinical Insights from a Large Commercial Insurance Database
Source: Dig Dis Sci. 2026 Apr 16;71(9):4400–5. doi: 10.1007/s10620-026-09905-7 (PMC13522055; doi:10.1007/s10620-026-09905-7)
Supplement: Supplementary file 1 [file 10620_2026_9905_MOESM1_ESM.docx]

**Supplemental Table 1: International Classification of Diseases, Tenth Revision, Clinical Modification (ICD-10-CM) and Current Procedural Terminology (CPT) codes used to identify conditions and procedures of interest**

| **Description** | **ICD-10-CM code(s)** |
| --- | --- |
| **Conditions** |  |
| Anxiety disorder | F40–F41 |
| Autoimmune inflammatory disease | G35, G70, K50, K51, L40, L93, M05, M06, M08, M33, M35.2, M45 |
| Bacterial intestinal infection, unspecified | A04.9 |
| Candidiasis |  |
| Candidal enteritis | B37.82 |
| Vulvovaginal candidiasis | B37.3 |
| Chronic pain | G89.2, G89.4 |
| Colectomy | Z90.49 |
| Depression | F32–F33 |
| Diabetes | E10–E13 |
| Dietary counseling and surveillance | Z71.3 |
| Dyslipidemia | E78 |
| Fibromyalgia | M79.7 |
| Gastroesophageal reflux disease | K21 |
| *Helicobacter pylori* | B96.81 |
| Hypertension | I10 |
| Hypothyroidism | E03 |
| Immunosuppression |  |
| HIV | B20, Z21 |
| Cancer | C00–C96, excluding C44 |
| Solid organ or stem cell transplant | T86, Z94 (excluding Z94.7), Z95.2, Z95.3 |
| Iron deficiency | E61.1 |
| Irritable bowel syndrome | K58 |
| Lyme disease | A69.2 |
| Malnutrition | E40-E46 |
| Nutritional anemia | D50-D53 |
| Overweight and obesity | E66 |
| Smoking (current or past) | F17, Z87.891 |
| Urinary tract infection | N39.0 |
| Vitamin D deficiency | E55 |
| **Signs and symptoms** |  |
| Abdominal distention | R14.0 |
| Abdominal pain | R10 |
| Abnormal weight gain | R63.5 |
| Abnormal weight loss | R63.4 |
| Constipation | K59.0 |
| Diarrhea | R19.7 |
| Fatigue or malaise | R53 |
| Insomnia | G47.0 |
| Nausea and vomiting | R11 |
| **Description** | **CPT code(s)** |
| Fungal stool culture | 87102, 87106 |
| Bacterial stool culture | 87045, 87046, 87075, 87076, 87077 |
| *Candida* antibody test | 86628 |
| Breath hydrogen or methane test | 91065 |
| Esophagogastroduodenoscopy | 00731, 00732, 00740, 00813, 43233, 43235–43259, 43266, 43270 |
